# Intermittent Exposure of Hypercapnia Suppresses Allograft Rejection via Induction of Treg Differentiation and Inhibition of Neutrophil Accumulation

**DOI:** 10.3390/biomedicines10040836

**Published:** 2022-04-01

**Authors:** Yuan-Sheng Tzeng, Yi-Jen Peng, Shih-En Tang, Kun-Lun Huang, Shi-Jye Chu, Shu-Yu Wu, Chia-Pi Cheng

**Affiliations:** 1 Graduate Institute of Medical Sciences, National Defense Medical Center, Taipei 114202, Taiwan; m6246kimo@yahoo.com.tw (Y.-S.T.); kun@ndmctsgh.edu.tw (K.-L.H.); 2 Division of Plastic Surgery, Department of Surgery, Tri-Service General Hospital National Defense Medical Center, Taipei 114202, Taiwan; 3 Department of Pathology, Tri-Service General Hospital, National Defense Medical Center, Taipei 114202, Taiwan; yijen0426@gmail.com; 4 Institute of Pathology and Parasitology, National Defense Medical Center, Taipei 114202, Taiwan; 5 Institute of Aerospace and Undersea Medicine, National Defense Medical Center, Taipei 114202, Taiwan; msetang@gmail.com; 6 Division of Pulmonary and Critical Care Medicine, Department of Internal Medicine, Tri-Service General Hospital, National Defense Medical Center, Taipei 114202, Taiwan; 7 Division of Rheumatology, Immunology and Allergy, Department of Internal Medicine, Tri-Service General Hospital, National Defense Medical Center, Taipei 114202, Taiwan; d1204812@mail.ndmctsh.edu.tw; 8 Department and Graduate Institute of Biology and Anatomy, National Defense Medical Center, Taipei 114202, Taiwan

**Keywords:** allogeneic skin graft, skin rejection, carbon dioxide, hypercapnia, regulatory T cells

## Abstract

Background: In the management of major burn wounds, allogeneic skin transplantation is a critical procedure to improve wound repair. Our previous works found that intermittent exposure to carbon dioxide leads to permissive hypercapnia (HCA) and prolongs skin allograft survival. However, the modulatory effects of HCA exposure on the immune system are not well understood. Objectives: Our purpose was to investigate how intermittent exposure to HCA can effectively reduce the immune reaction to allogeneic skin graft rejection. Methods: A fully major histocompatibility complex-incompatible skin transplant from BALB/c to C57BL/6 mice model was utilized. Immune cells from splenic and draining lymph nodes were analyzed by flow cytometry. Serum proinflammatory cytokines were analyzed by ELISA. Results: Serum levels of IFN-γ, IL-2, IL-6, and TNF-α were significantly decreased in the HCA group. Additionally, the percentage of CD8+ cells in draining lymph nodes was significantly lower in HCA than in the control group. Moreover, the generation rate of FoxP3+ regulatory T cells (Tregs) from spleen naïve CD4+ T cells was increased by intermittent exposure to carbon dioxide. The infiltrated neutrophils were also eliminated by HCA. Taken together, we concluded that intermittent hypercapnia exposure could effectively suppress skin rejection by stimulating Treg cell generation and suppressing immune reactions.

## 1. Introduction

Early excision and grafting have been the standard treatment for large burns [1,2]. Since an autograft is derived from the patient’s own tissue, there is no risk of rejection [3]. However, the limitation of harvest sites and amount of graft material and additional surgical sites for the grafts lead to an inability to perform autografts. A method to overcome the limitation of donor tissue for skin grafting with an autograft is to use allografts [3]. Although allograft coverings will eventually cause inflammation and immune rejection [4], they can provide at least one week of coverage, reducing fluid loss, diminishing metabolic demand, developing a well-developed wound bed, and increasing the success rate of autologous skin transplantation [5,6,7]. Generally, allograft removal and replacement procedures were repeated every 3–4 weeks until the wound heals [8,9]. Therefore, finding the best way to prolong skin allograft survival, reducing multiple surgical procedures, protecting the wound from infection, and decreasing mortality are the most important strategies for large burn transplantations.

Acute cellular rejection is a type of organ dysfunction initiated by cell-mediated immunity in skin transplant recipients. During acute cellular rejection, although T cells have a critical role and are involved in the modulation of antigen-specific responses, an up-regulation of pro-inflammatory mediators in the allograft occurs before the T cell response. Additionally, an imbalance between pro-inflammatory (interleukin-6, tumor necrosis factor-α, and interferon-γ) and anti-inflammatory (interleukin-4 and interleukin-10) cytokines leads to an immune response resulting in acute cellular rejection [10,11,12,13]. Moreover, these cytokines attract the innate immune system, such as neutrophils, macrophages, and NK cells, which rapidly infiltrate the graft site and facilitate immune rejection [14,15,16]. The pharmaceutical strategy of immunosuppressive drugs can selectively inhibit the activities of T lymphocytes, cytokines (interleukin-6, tumor necrosis factor-α, interferons, interleukin-2), and other substances from achieving curative effects [17,18,19]. However, long-term administration will suppress normal immune function and increase the risk of infection [9,20]. Therefore, finding a way to reduce, not abrogate, the immune response would be more suitable for patients with allogeneic skin transplantation.

Permissive hypercapnia (HCA) accompanying low tidal volume ventilation in lung injury and acute respiratory failure is an accepted clinical practice in which carbon dioxide is elevated [21,22]. Previous studies have documented the protective effects of HCA induced by adding inspired carbon dioxide in animal models of lung injury, including in free radicals, sepsis, ischemia-reperfusion, and acute cellular rejection [10,23,24,25,26]. Our earlier study demonstrated that HCA prolonged skin allograft survival by suppressing serum levels of TNF-α and NF-κB activation in draining lymph nodes [27]. Based on our previous findings, we hypothesize that HCA might modulate T lymphocyte responses and help decrease the chances of acute cellular rejection.

## 2. Materials and Methods

### 2.1. Animals

In this study, 8- to 12-week-old male C57BL/6 and BALB/c mice from BioLasco Taiwan Co., Ltd., Yi-Lan, Taiwan, were bred in a pathogen-free environment and used for skin graft experiments. All animals were cared for according to the guidelines of the National Institutes of Health (National Academy Press, Washington, DC, USA, 1996), and all experimental protocols were approved by the National Science Council and Animal Review Committee of the National Defense Medical Center.

### 2.2. Skin Graft Experiments

The dorsal skin of BALB/c mice was transplanted onto the C57BL/6 mice under sterile conditions (allogeneic transplantation). The dorsal skin of C57BL/6 mice was transplanted onto the C57BL/6 mice (syngeneic transplantation) as the sham control. On the experiment day, the mice were anesthetized with a mixture of Zoletil 50 (Virbac Laboratoire, Viguie, France, 0.1 mL/100 g) and Rompun (Bayer Korea, Seoul, Republic of Korea, 0.02 mL/100 g). Full-thickness trunk skin (1 × 1.5 cm^2^) from the donor mice was promptly grafted into a graft bed on the back of the recipient. The graft was fixed to the graft bed with 10 interrupted sutures of 5-0 monofilament nylon thread (Dermalon; Davis and Geck, St. Louis, MO, USA). No dressings or antibiotics were used.

### 2.3. Experimental Protocols

The hypercapnic acidosis protocol was performed as described previously [27]. The mice were randomly assigned to the sham control group (*n* = 20), skin graft group (air group) (*n* = 20), or HCA and skin graft group (*n* = 20). HCA-treated mice were exposed to 5% CO_2_ in the air daily for one hour from day 0 to day 7 after transplantation. The target pH and PCO_2_ values were around 7.2 and 70 mmHg. Specimens were collected on days 1, 3, and 7 after the operation.

### 2.4. Evaluation of Skin Rejection

In brief, necrotic areas were evaluated according to the previous study by Xiangli et al. Score levels were well defined by the percentage of necrotic area and divided into 6 scores (0–5) [28].

### 2.5. Measurement of Cytokines in Serum

Serum obtained from blood samples was frozen at −80 °C, then thawed to room temperature when analyzed. The levels of IFN-γ, IL2, IL-4, IL-10, IL-6 and TNF-α were analyzed by using ProcartaPlex multiplex preconfigured panels according to the manufacturer’s instructions (eBioscience, Thermo Fisher Scientific, San Diego, CA, USA). The levels of TGF-β1 and CCL-2 in serum were determined using a commercially available mouse ELISA kit (R&D Systems Inc., Minneapolis, MN, USA).

### 2.6. Flow Cytometry Analysis

Cells from the spleen or lymph node after transplantation were harvested and stained with anti-CD8 (FITC), anti-CD4 (FITC), and anti-Foxp3 (PE) antibody (eBioscience, Thermo Fisher Scientific, San Diego, CA, USA). Cells from Treg differentiation were stained with anti-CD4 (FITC) and anti-Foxp3 (PE) antibodies. All experiments were in accordance with the manufacturer’s instructions. The fluorescence labeling was measured with the BD FACSCalibur flow cytometry system equipped with fluorescence detectors, and bandpass filters, 530 nm for FITC and 585 nm for PE/PI (BD Biosciences, Franklin Lakes, NJ, USA), and the data were analyzed using CellQuest Pro software.

### 2.7. Western Blot Analysis

Western blotting analysis was performed as in our previous study [27]. Primary antibodies against CD4 (diluted 1:400; Bioss Inc., Woburn, MA, USA), FoxP3 (diluted 1:1000; Merck Millipore Co, Burlington, MA, USA) and β-actin (1:10,000, Sigma Chemical Company, St. Louis, MO, USA) were incubated overnight at 4 °C. The blots were then washed in PBST 3 times for 10 min. Blots were incubated with HRP-conjugated goat anti-rabbit IgG (1:20,000) and goat anti-mouse IgG (1:50,000) for 1 h at room temperature, then washed 3 times in PBST for 10 min. Bands were visualized using enhanced chemiluminescence reagents and by exposing the blot to a UVP ChemiDoc-It Imaging System. The ratios of the band intensities were calculated.

### 2.8. Naïve T Cell Isolation and Treg Differentiation

Naïve CD4^+^ T cells were isolated from the spleen by negative selection using Dynabeads Untouched Mouse T Cells Kit (Invitrogen, Thermo Fisher Scientific, Carlsbad, CA, USA) according to the manufacturer’s instructions. Purified naïve CD4^+^ T cells were used in the Treg cell differentiation assay by the commercially available Mouse Treg Cell Differentiation Kit (R&D Systems Inc., Minneapolis, MN, USA). The 8 × 10^5^ cells were added to the wells at a 1 mL/well volume. Cells were cultured at 37 °C with 5% CO^2^ for 3 days and analyzed by flow cytometry. The HCA group was daily cultured at 37 °C at 10% CO_2_ for one hour and backed to 37 °C at 5% CO^2^.

### 2.9. Histological Assessment

After fixation, the skin grafts from each time point of the experiment were stained with H&E and analyzed using a VENTANA DP 200 slide scanner (which provided the VENTANA Virtuoso Software).

### 2.10. Immunohistochemical Analysis

Next, 4-μm paraffin embedding microsections were stained with primary monoclonal anti-CD4, anti-Foxp3 and anti-Ly6G antibodies for 60 min after deparaffinization and antigen retrieval. Subsequently, the slides were washed and sequentially incubated with rat tissue-specific horseradish peroxidase-polymer anti-mouse antibody (Nichirei Corporation, Tokyo, Japan) for 30 min. The horseradish peroxidase was then reacted with DAB substrate for three minutes, and the sections were then counterstained with hematoxylin.

### 2.11. Neutrophil Isolation and Migration Assay

Mouse neutrophils were isolated as described previously (Oh, Siano & Diamond, 2008). Neutrophil chemotaxis was performed by using a 3 μm pore Transwell system (Corning Costar) with the MIP-2 (1 μg/mL) incubation at 37 °C for 60 min. In brief, a cell suspension containing 1 × 10^6^ freshly isolated neutrophils in a total volume of 150 μL serum-free RPMI 1640 medium was added to the upper chamber of the transwell. After incubation, cells in the lower chamber were harvested and stained with BCECF-AM (Invitrogen, Thermo Fisher Scientific, Carlsbad, CA, USA) or counted by ADAM MC Auto Cell Counter.

### 2.12. Statistical Analysis

The data are expressed as mean ± SD. Statistical differences between group means were determined with one-way and two-way ANOVA, followed by a post hoc comparison using the Bonferroni post-test. Significance was determined at the *p* < 0.05 level.

## 3. Results

**Figure 1:**
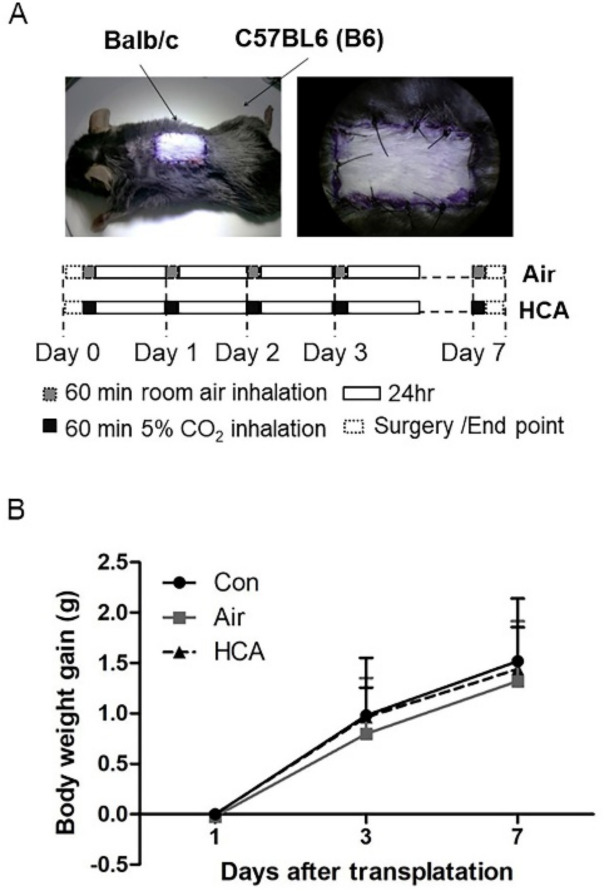
Experimental protocol. (**A**) Hypercapnic acidosis (HCA) protocol and (**B**) body weight gain in mice after transplantation.

The experimental flow chart is shown in Figure 1A. The skin-allografted mice were exposed to hypercapnic acidosis or air one hour a day for seven consecutive days. There was no significant difference between each group in body weight changes of mice from day 1 to 7 post-transplantation (Figure 1B).

### 3.1. Effects of HCA on Necrotic Levels in Skin Graft Mice

**Figure 2:**
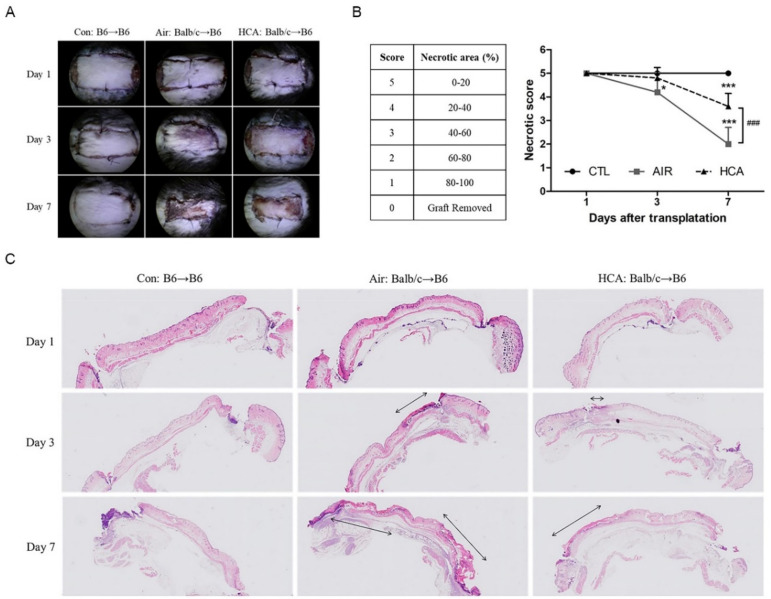
Macroscopic images of skin grafts in mice after transplantation. (**A**) Representative photographs are shown of recipient mice after receiving skin allografts. (**B**) Statistical analysis of the necrotic levels of grafts from day 1 to day 7 post-transplantation. (**C**) Histological appearance of skin grafts shown by a representative micrograph of skin tissue (10× magnification). The double arrow indicates the area of skin necrosis (*n* = 5 mice/day/group). * *p* < 0.05 and *** *p* < 0.001 compared with Con group; ^###^ *p* < 0.001 compared with Air group.

C57BL/6 mice were transplanted with BALB/c dorsal skin. After transplantation, necrotic areas of skin grafts were monitored daily by photographs until day 7 post-transplantation (Figure 2A). Mice were evaluated by 6 different score levels (0–5) according to the necrotic area of the skin graft. Our results showed that skin grafts’ necrotic levels were markedly lower in the HCA-treated group than in the air group on day 7 (Figure 2B). Additionally, the histopathology of graft skins showed the epidermis of necrosis areas from day 3 to day 7 were dramatically decreased in the HCA-treated mice. (Figure 2C).

### 3.2. Effects of HCA on Serum Cytokine Expression in Skin Graft Mice

**Figure 3:**
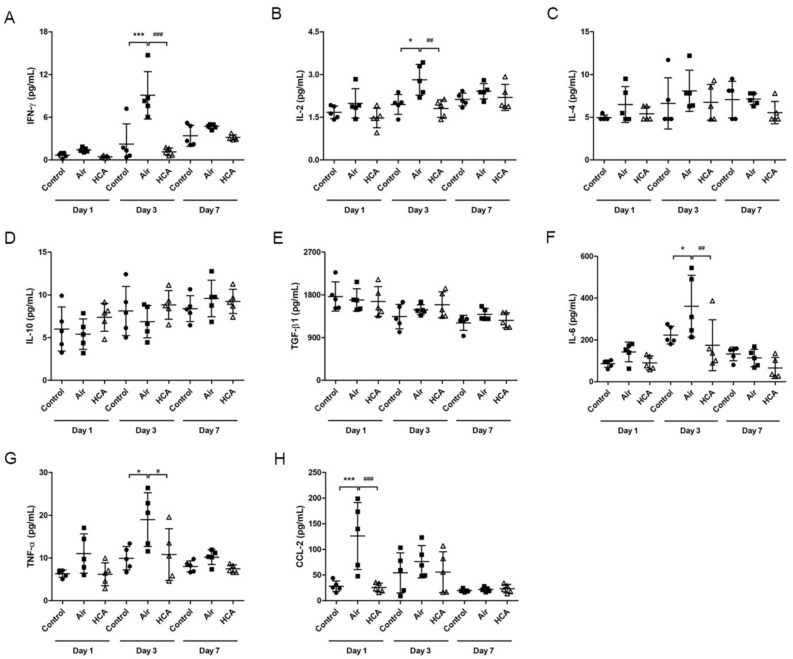
Serum cytokine levels in mice transplanted with allogenic skin transplants. The serum concentration of IFN-γ (**A**), IL-2 (**B**), IL-4 (**C**), IL-10 (**D**), TGF-β1 (**E**), IL-6 (**F**), TNF-α (**G**), and CCL-2 (**H**) in mice were evaluated by ELISA, respectively (*n* = 5 mice/group). * *p* < 0.05; *** *p* < 0.001 compared with the control group; ^#^ *p* < 0.05; ^##^ *p* < 0.01; ^###^ *p* < 0.001 compared with the Air group.

To further examine the necrotic suppression of HCA on grafted skin, we analyzed the cytokine levels in serum at different time points by ELISA. Our result shows that most of the cytokines were elevated at their peak on day 3 after transplantation. Notably, the serum levels of Th1 cytokines, IFN-γ and IL-2 were significantly lower in HCA-treated mice compared with the air group (Figure 3A,B). The HCA-treated mice demonstrated only one-tenth as much serum IFN-γ as air group mice (1.07 pg/mL vs. 10.02 pg/mL) and approximately 63% of serum IL-2 as air group mice. Furthermore, the proinflammatory cytokines IL-6 and TNF-α and chemokine CCL2 were also significantly lower in HCA-treated mice as compared with the air group (Figure 3F–H). However, the serum levels of Th2 cytokines, IL-4, IL-10 and TGF-β, were not significantly different in each group (Figure 3C–E).

### 3.3. Effects of HCA on T Cell Population in Skin Graft Mice

**Figure 4:**
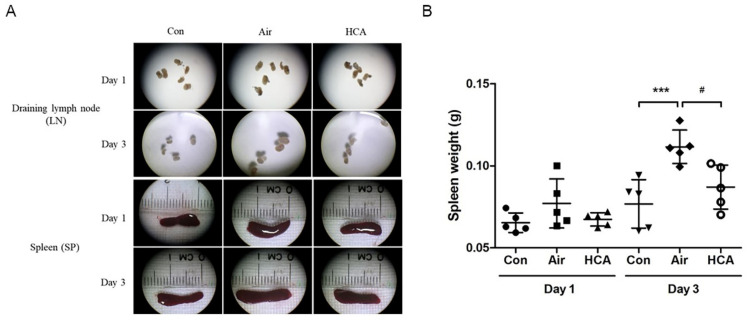
Macroscopic images of draining lymph nodes and spleens after transplantation. (**A**) The representative photographs of draining lymph nodes and spleen size are shown for recipient mice after receiving skin allografts. (**B**) Spleen weight was measured on days 1 and 3 post-skin graft (*n* = 5 mice/group). *** *p* < 0.001 compared with Con group; ^#^ *p* < 0.05 compared with Air group.

**Figure 5:**
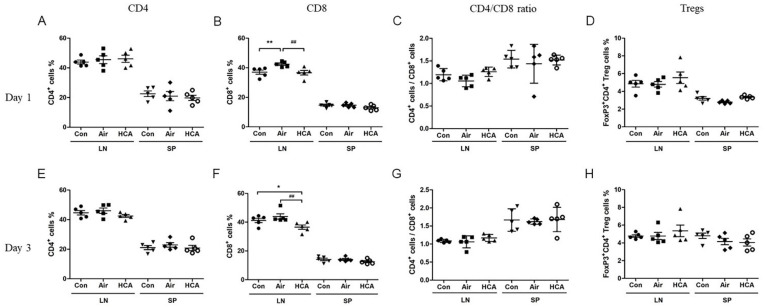
Immunophenotypes of draining lymph nodes and spleen T cells. The percentage of CD4^+^ (**A**), CD8^+^ (**B**), CD4/CD8 ratio (**C**), and regulatory T cells (Treg) (**D**) on day 1 and CD4^+^ (**E**), CD8^+^ (**F**), CD4/CD8 ratio (**G**), and regulatory T cells (Treg) (**H**) on day 3 after allogeneic transplantation were analyzed by flow cytometry, respectively. (*n* = 5 mice/group). * *p* < 0.05; ** *p* < 0.01 compared with Con group; ^##^ *p* < 0.01 compared with Air group.

**Figure 6:**
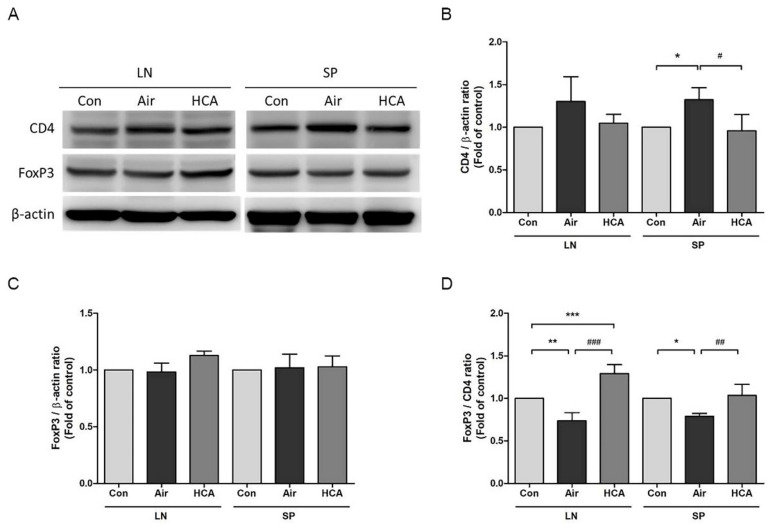
Expression levels of CD4 and FoxP3 protein in draining lymph nodes and spleen. (**A**–**D**) Protein expression levels of CD4 and FoxP3 from draining lymph nodes and spleen extracts on day 3 after transplantation were quantified by Western blotting. A representative result of at least three independent experiments is shown. (*n* = 3 mice/group). * *p* < 0.05; ^#^ *p* < 0.05; ** *p* < 0.01; ^##^ *p* < 0.01; *** *p* < 0.001 compared with Con group; ^###^ *p* < 0.001 compared with Air group.

**Figure 7:**
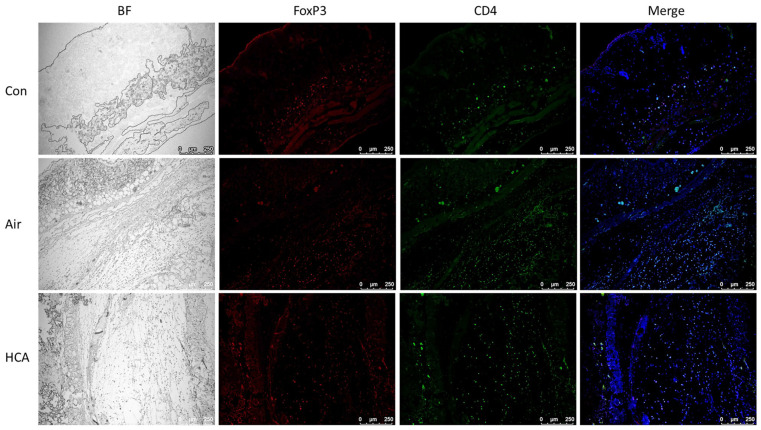
Immunofluorescence staining of FoxP3 and CD4 in skin grafts. The skin grafts from each group were performed with bright field (BF) or staining with anti-FoxP3 antibody (red), anti-CD4 antibody (green), and DAPI (blue) on day 3 after transplantation. A representative micrograph and merge image were shown (100× magnification).

To further analyze which T cell population was dominantly affected by HCA, we isolated the cells from draining lymph nodes and spleens in grafted mice on days 1 and 3 after transplantation. Interestingly, the phenomenon of enlarged lymph nodes and splenomegaly was observed in the air group on day 3 but was suppressed by HCA treatment (Figure 4A). Allografted mice in the air group underwent a 1.6-fold increase in spleen weight compared with a 1.1-fold increase in the HCA-treated mice on day 3 (Figure 4B). Furthermore, the percentage of CD8^+^ T-cells in lymph nodes was decreased in the HCA-treated group on days 1 and 3 as compared with control groups (sham control:air control:HCA group = 37.0 ± 3.2%: 42.4 ± 1.8%: 36.6 ± 3.5%, *p* < 0.01; sham control:air control:HCA group = 41.2 ± 3.5%: 43.9 ± 4.3%: 36.5 ± 3.4%, *p* < 0.05) (Figure 5B,F). Moreover, the HCA group tended to have a higher percentage of FoxP3^+^CD4^+^ T cells in lymph nodes and spleens, but the statistical analysis was not significant (Figure 5D,H). The percentage of CD4^+^ T-cells on both days 1 and 3 was not affected by HCA compared with the control groups (Figure 5A,E). Otherwise, to analyze the protein expression of CD4 and FoxP3 in draining lymph nodes and spleens on day 3 post-transplantation found that the FoxP3/CD4 ratio in lymph nodes and spleens was significantly increased (*p* < 0.001, *p* < 0.01) in the HCA group as compared with the air group (Figure 6 and Figure 7).

### 3.4. Effects of HCA on Differentiation of Regulatory T Cells from Splenic-Isolated Naïve CD4^+^ T Cells

**Figure 8:**
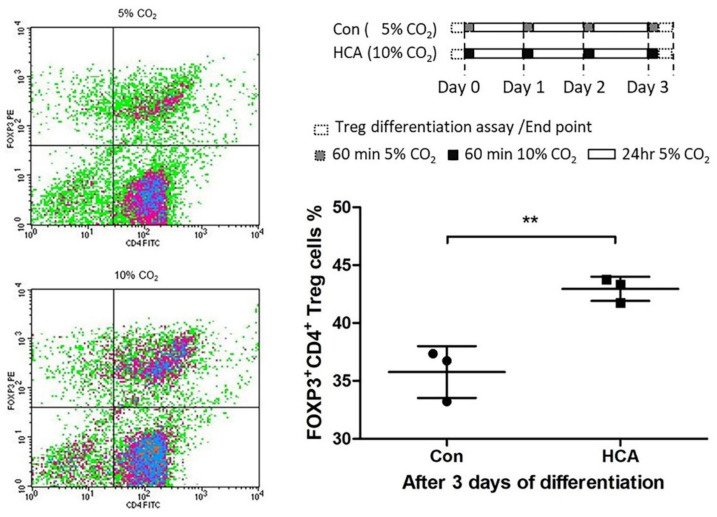
Effect of HCA on de novo generation of Tregs from spleen-isolated naïve CD4^+^ T cells. Splenic-isolated naïve CD4^+^ T cells were used for inducing Treg differentiation. After 3 days of culture, the Treg cells’ percentage was analyzed by flow cytometry. Error bars represent the SD from three independent experiments; ** *p* < 0.01.

Previous studies reported that regulatory T cells play a modulatory role in allograft transplantation in CD4/8 T cell proliferation and activation. To confirm and clarify the trend of FoxP3^+^CD4^+^ T cells, enhancement was induced by HCA exposure, and naïve CD4^+^ T cells were isolated and used for further in vitro Treg differentiation experiments. Our results show that the percentage of Treg cells was significantly increased in the HCA-treated group as compared with untreated cells (Figure 8; Con: HCA = 35.77 ± 2.23%: 42.95 ± 1.05%, *p* < 0.01).

### 3.5. Effects of HCA on Neutrophil Infiltration in Skin Graft Mice

**Figure 9:**
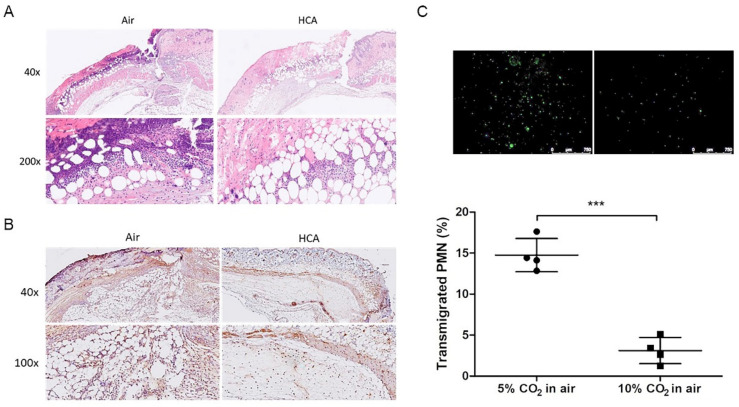
Effects of HCA on neutrophil recruitment in mice. (**A**) Histological appearance of skin grafts on day 3 after transplantation was shown by a representative micrograph (40× and 200× magnification). (**B**) The immunohistochemical staining (200× magnification) of Ly6G expression in the grafts. (**C**) A neutrophil migration assay was performed by the MIP-2 (1 μg/mL) chemoattractant. Migrated neutrophils were stained (green) and evaluated by fluorescence microscopy (10×; white bar = 750 μm). Error bars represent the SD from four independent experiments; *** *p* < 0.001.

Based on a previous study, the immune response to a graft depended on not only the adaptive immune responses but also the primary innate immune allorecognition [14]. As shown in Figure 3H, the monocyte chemoattractant protein-1 (CCL-2/MCP-1), a chemokine related to attracting monocytes, dendritic cells, or T cell infiltration, was significantly decreased in HCA-treated mice on day 1 to compare with air group mice post-transplantation. The allografted skin of mice in the air group showed progressive inflammatory cell infiltration and was markedly eliminated by the HCA exposure (Figure 9A). To further check the innate immunity involved in this allografted transplantation, infiltrated neutrophils were detected by immunohistochemistry staining. Our results showed that the Ly6g^+^ neutrophils were dominantly infiltrated on day 3 after transplantation in the air group but were reduced by HCA exposure (Figure 9B). Moreover, to clarify whether HCA could directly influence neutrophil migration, we isolated the neutrophils from the blood of B6 mice for a transmigration assay. Our results showed that HCA significantly reduce neutrophil migration from the upper to lower chamber after 1 h of 10% CO_2_ treatment (Figure 9C; 5% CO_2_: 10% CO_2_ = 14.75 ± 2.03%: 3.13 ± 1.16%, *p* < 0.001).

## 4. Discussion

Finding an effective way to reduce immune rejection is one of the most important strategies for supporting clinical transplantations. Our research found that intermittent exposure to highly concentrated carbon dioxide leads to hypercapnia, which can effectively delay the rate of immune rejection and necrosis of allogeneic skin grafts. Our findings are consistent with previous studies that revealed the protective effects of inhaled carbon dioxide in animal models of endotoxin-induced lung injury [23,24] and ischemia-reperfusion injury [10,25,26,29]. In clinical practice, HCA inhibits local and systematic inflammation and improves respiratory function after one-lung ventilation in lobectomy patients without severe complications [30]. However, HCA treatment still has the risk of some diseases, such as pneumonia infection. Long-term (>48 h) exposure to HCA has been shown to impair the host response to an invaded pathogen, promote bacterial proliferation, and lead to aggravating lung injury, but short-term (<6 h) exposure did not [31,32,33]. This means the timing and duration of the HCA administration were critical to shifting the balance in favor of benefits. Our findings supported that intermittent inhaled carbon dioxide was a potential strategy to block acute cellular rejection in allogeneic transplantation.

Allograft rejection with a complicated mechanism is involved in innate and adaptive immunity. Most researchers agree that rejection is induced by direct or indirect ways of antigen-presenting to T cells [34]. Activated T cells amplify the immune reaction by secreting cytokines to activate, expand, and/or recruit other effector cells, such as macrophages, neutrophils, CD8^+^ T cells, and B cells [34,35,36]. For example, IFN-γ, a cytokine produced by activated T or NK cells, acts both on the immune system and the graft itself, which lies in its ability to induce major histocompatibility complex (MHC) [12]. IL-2 is another cytokine which acts functionally on T cell proliferation and differentiation, modulating complex effects on allograft rejection [13]. These two cytokines initiate T-cell growth and survival during the immune response and then reinforce the Th1 response with positive feedback in allograft rejection [12,13]. In this regard, our results showed that HCA significantly reduced serum concentrations of IL-2, IFN-γ, and TNF-α (Figure 3). Furthermore, the accumulation of excess lymphocytes in the draining lymph nodes and spleen was reduced by HCA treatment (Figure 4). Additionally, an analysis of the immunophenotypes of T cells in draining lymph nodes has found that CD8^+^ T cells were suppressed in the HCA group. Moreover, based on previous studies, a lower CD4/CD8 ratio has been associated with a higher possibility of allograft rejection [26,37]. However, our results showed that the CD4/CD8 ratio increased in the HCA group on day 1 and day 3. There was no significant difference in the ratio of CD4 to CD8 between groups, which may indicate that HCA suppressed both CD4^+^ and CD8^+^ T cells at each time point (Figure 5).

Studies conducted in recent decades have pointed out that in a subset group of CD4^+^ T cells, regulatory T cells were a key modulator of allograft rejection for maintaining self-tolerance and preventing autoimmune disease [38,39,40,41,42,43]. Interestingly, we are the first group to find that HCA can directly modulate regulatory T cell differentiation. Our results showed that HCA treatment raised a trend of CD4^+^FoxP3^+^ regulatory T (Treg) cell populations in vivo (Figure 5D,H). Furthermore, isolated naïve T cells for Treg cell differentiation were also increased in the HCA-treated group in vitro (Figure 8). The expression levels of CD4 and FoxP3 protein in draining lymph nodes, spleens and grafts have shown an increased FoxP3/CD4 ratio in the HCA group compared with the air group (Figure 6 and Figure 7). These indicated that HCA induced Treg cell differentiation to modulate T lymphocyte immune responses and to help decrease acute cellular rejection.

In addition, innate immune cells also participated in the orchestration of allograft rejection after transplantation. Innate immune cells such as neutrophils are recruited to the graft site early after reperfusion and subsequently promote allograft rejection [41,44,45,46,47]. In our results, the allografted skin of mice showed progressive inflammatory cell and Ly6g^+^ neutrophil infiltration in the air group, and this phenomenon was markedly reduced in the HCA-treated skin (Figure 9A,B). Histologic evaluation based on pathologic necrotic scores of allograft rejection on day 3 demonstrated that HCA showed a significant reduction in the rejection score compared with the air group (Figure 2). Moreover, an in vitro neutrophil transmigration assay also showed the significant suppression abilities of HCA exposure (Figure 9C). Our data supported that the role of participating neutrophil infiltrations in allograft rejection would be greatly improved by HCA; similarly, HCA may be useful in the as suppression of acute lung allograft rejection.

## 5. Conclusions

**Figure 10:**
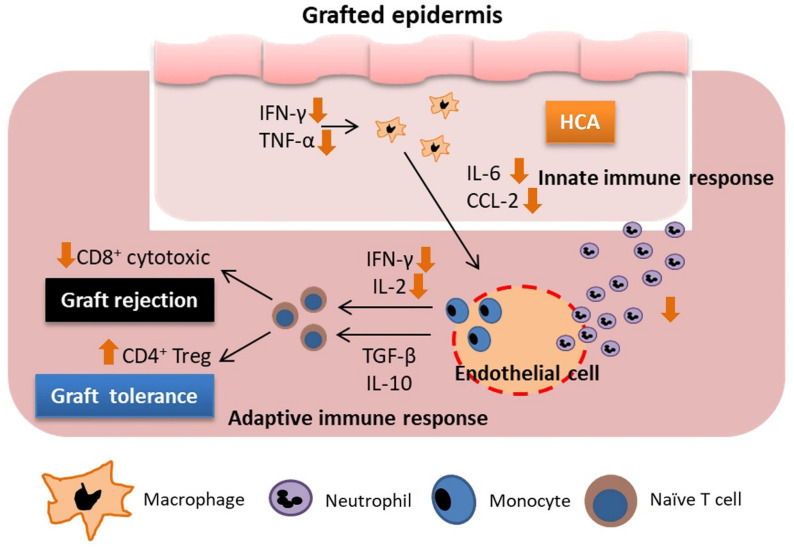
The diagram of the effects of HCA on the regulation of innate and adaptive immune cells in allograft rejection.

Our study supported that hypercapnia effectively ameliorates acute cellular rejection in the skin of a mouse acute allograft rejection model by suppressing the expression of proinflammatory cytokines and neutrophil infiltration, inhibiting T cell activation and accumulation, and inducing Treg cell differentiation (Figure 10). This provides a theoretical basis for the development of novel strategies against acute cellular rejection after skin transplantation.

## Data Availability

All data, models, and code generated or used during the study appear in the submitted article.

## Declaration of Transparency and Scientific Rigour

This Declaration acknowledges that this paper adheres to the principles for transparent reporting and scientific rigour of preclinical research as stated in the BJP guidelines for Design & Analysis, Immunoblotting and Immunochemistry, and Animal Experimentation, and as recommended by funding agencies, publishers and other organizations engaged with supporting research.
